# Viral loads in nasopharyngeal aspirates and tracheal aspirates among children hospitalized with invasive ventilation for human adenovirus pneumonia

**DOI:** 10.1186/s12985-021-01711-z

**Published:** 2021-11-30

**Authors:** Le-Yun Xie, Sai-Zhen Zeng, Tian Yu, Xian Hu, Tao Wang, Le Yang, Li-Li Zhong, Jin-Song Li, Zhao-Jun Duan, Bing Zhang

**Affiliations:** 1 Hunan Provincial People’s Hospital (The First Affiliated Hospital of Hunan Normal University), 61 Jie-Fang west road, Fu-Rong District, Changsha, 410005 China; 2 Hunan Provincial Key Laboratory of Pediatric Respirology, Changsha, 410005 China; 3 MOH Key Laboratory for Medical Virology and Viral Diseases, National Institute for Viral Disease Control and Prevention, China CDC, Beijing, 100052 China

**Keywords:** Human adenovirus, Mechanical ventilation, Nasopharyngeal aspirate, Tracheal aspirate, Viral load

## Abstract

**Purpose:**

To evaluate viral loads in children with human adenovirus (HAdV) pneumonia at different stages of disease and compare the viral load between upper and lower respiratory tract samples.

**Methods:**

We prospectively enrolled children who required invasive ventilation for HAdV pneumonia. Nasopharyngeal aspirate (NPA) and tracheal aspirate (TA) samples were collected throughout the entire period of invasive ventilation. Viral detection and quantification were performed using quantitative real-time polymerase chain reaction.

**Results:**

Ninety-four children were enrolled. The median age of the children was 12.0 months (IQR: 11.0–24.0), and > ninety percent of patients were aged between 6 and 59 months. Seven hundred and nine paired NPA-TA samples were collected. The median viral loads of the NPA and TA samples were 7.31 log10 and 7.50 log10 copies/mL, respectively. Viral loads generally decreased steadily over time. The median viral load after 1, 2, 3, and > 3 weeks of the disease course was 8.65, 7.70, 6.69, and 5.09 log10 copies/mL, respectively, in NPA samples and 8.67, 7.79, 7.08, and 5.53 log10 copies/mL, respectively, in TA samples. Viral load showed a significant negative correlation with time since symptom onset in both NPA samples (Spearman r =  − 0.607, *P* = 0.000) and TA samples (Spearman r =  − 0.544, *P* = 0.000). The predicted duration of HAdV shedding was 60.17 days in the NPA group and 65.81 days in the TA group. Viral loads in NPA and TA from the same subjects correlated well with each other (R^2^ = 0.694). HAdV loads in NPA and TA were most comparable during the early phase of infection (95% limits of agreement, − 1.36 to 1.30 log10 copies/mL, R^2^ = 0.746). Variation increased during the late phase of infection (*i.e*., in follow-up samples), with viral loads remaining significantly higher in TA than NPA.

**Conclusions:**

In children with HAdV pneumonia, viral loads in both NPA and TA steadily decreased during the course of the disease, and the predicted duration of viral shedding was more than 2 months. The HAdV DNA load of NPA is highly correlated with that of TA, especially in the initial phase of infection.

## Background

Human adenoviruses (HAdVs) are important pathogens in paediatric pneumonia [1, 2]. Although the prevalence of adenovirus-associated pneumonia (10–20%) is lower than that of respiratory syncytial virus, this type of infection can induce severe and fatal necrotizing pneumonia (especially HAdV-3 and 7) [3–7]. It is currently unclear why most immunocompetent children experience relatively mild respiratory symptoms, whereas a small subgroup of children develops respiratory failure necessitating mechanical ventilation in paediatric intensive care units (PICUs) [8–10]. Quantitative HAdV detection is critical for elucidating the relationship between HAdV infection and the severity of lower respiratory tract infection [7, 11]. In addition, the trend of viral loads over time provides insight into the risk of disease—persistent or rising viral replication is suggestive of progressive or disseminated disease [12]. Monitoring the kinetics of viral shedding can reveal disease and infection status and provide a basis for adjusting treatment and infection-control strategies.

One of the most important initial steps in measuring the HAdV DNA load consists of selecting the appropriate sample for quantification. According to studies, secretions from the lower respiratory tract of children with pneumonia are preferred over upper respiratory tract samples for detection of HAdV by real-time PCR because they come from the site of infection [13]. However, the collection of lower respiratory tract samples from children remains a challenge and is sometimes considered unethical. Nasopharyngeal aspirate (NPA) samples are often used in place of lower respiratory tract samples for virus detection in patients with acute lower respiratory tract infection [14, 15]. However, HAdV can be detected by PCR in the NPA of healthy, asymptomatic children [16, 17]. In addition, adenovirus nucleic acid has been found in the tonsil and adenoid tissues of young children undergoing routine tonsillectomies [18]. These studies suggest that confirming a positive result for HAdV from an NPA specimen is challenging. Consistency between results from NPA and bronchoalveolar lavage fluid has been reported [19] and HAdV positivity in nasopharyngeal swabs were associated with lower respiratory tract infection [20]. No studies have compared the viral loads in NPA and tracheal aspirate (TA) samples in children with HAdV pneumonia. Therefore, we enrolled children diagnosed with HAdV pneumonia who required mechanical ventilation and observed the viral load dynamics of NPA and TA over the course of the disease to explore the correlation between viral load and clinical progress.

## Methods

### Patients

The patient’s parents or guardians provided written informed consent for participation. Children admitted to PICU of Hunan Provincial People’s Hospital (Changsha, Hunan Province, China) from September 2017 to September 2019 were eligible to participate in this study. The inclusion criteria were as follows: (1) Patients with a diagnosis of severe community-acquired pneumonia. Severe community-acquired pneumonia is defined as a diagnosis of community- acquired pneumonia with one of the following conditions [21]: poor general condition, refusal to eat, or dehydration, impaired consciousness, increased respiratory rate (≥ 70 times/min in infants, > 50 times/min in older children), cyanosis, dyspnea (moaning, nasal stirring, chest indrawing), ≥ 2/3 pulmonary infiltration or involvement of multiple lobes, pleural effusion, pulse oxygen saturation ≤ 0.92, extrapulmonary complications. (2) HAdV load of > 1 × 10^2^ copies/mL in NPA at admission. (3) Invasive mechanical ventilation. (4) No history of immunodeficiency, organ or stem cell transplantation, treatment with systemic glucocorticoids over 12 weeks, or treatment with other immunosuppressants. The exclusion criteria were as follows: (1) HAdV load of ≤ 1 × 10^2^ copies/mL in NPA. (2) Duration of invasive ventilation < 3 days. (3) Withdrawal from the study on guardian request. (4) Unknown HAdV type.

### Specimen collection

NPA and TA samples were taken simultaneously after invasive ventilation. The samples were routinely collected at 8.00–10.00 am daily until the day of extubation. When paired sampling of NPA and TA specimens was not possible due to insufficient mucus in the nasopharynx or the trachea, a second attempt to obtain the sample was made shortly after the first attempt (usually within 2 h). If the second attempt was also unsuccessful, the pairing was considered “failed”.

Respiratory secretions were aspirated by a standardized protocol directly from the nasopharynx and trachea in a sterile manner by a specialist PICU staff and were not diluted [15, 22]. NPA was collected first, using a disposable catheter connected to a mucous trap. The specimens were transported under controlled temperature conditions, as stipulated in the standard operating procedure [15]. The samples were placed on ice within 1 h of collection and stored at − 80 °C within 24 h. Prior to extraction, samples were digested in 1:1 dithiothreitol and incubated at ambient temperature to degrade mucus. After processing, NPA/TA specimens were subsequently stored at − 80 °C for real-time PCR testing; a portion (0.2 mL) was shipped in batches on dry ice to the China Center for Disease Control and Prevention for adenovirus typing.

### Laboratory experiments

Magnetic beads (Multi-Type Sample DNA/RNA Extraction-Purification Kit; Sansure Biotech Inc., Changsha, China) were used to extract DNA from 200 μL of NPA and TA samples following the protocol of the manufacturer. The extracted DNA was eluted in 50 μL of elution buffer and the eluted solution was stored at − 80 °C until required.

The hexon gene of HAdV was amplified by quantitative real-time PCR (SLAN 96S Real-Time PCR System, Shanghai Hongshi Medical Technology Co. Ltd., Shanghai, China) using a Respiratory Adenovirus DNA Diagnostic Kit (Sansure Biotech Inc., Changsha, China) according to the manufacturer’s instructions. The PCR reaction comprised 43 μL of a buffer, 2 μL of Taq enzyme (5 U), 0.2 μL each of four primers (50 M), 0.2 μL each of two probes (50 M), and 5 μL of DNA. The kit also contained plasmid DNA of a defined HAdV copy number as a standard. The target-sequence copy number was inversely proportional to the cycle threshold. For positive samples, adjusted Ct values were calculated by correcting the Ct values for the initial sample dilution, and the corrected Ct values were converted to copies/mL using standard curves. An HAdV DNA load of < 1 × 10^2^ copies/mL was considered negative. Glyceraldehyde 3-phosphate dehydrogenase was used as the internal control. Intrasample variation was assessed by dividing material from 10 NPA samples and 10 TA samples into three aliquots, followed by DNA isolation and real-time PCR separately for each aliquot. The precision test showed excellent within -NPA and -TA reproducibility; the coefficient of variation of the detection result Ct value was ≤ 5%. According to Lu and Erdman, the hypervariable region (1–6) of loop 1 of the hexon protein was sequenced for HAdV typing [23].

### Statistical analysis

Statistical tests were performed using R software (version 3.6.2; R Development Core Team, Vienna, Austria). A *P* value of < 0.05 was considered indicative of significance. The quantitative PCR data were log10 transformed. Median values are reported with interquartile range (IQR). Statistical comparisons between the NPA and TA groups were evaluated by two-tailed paired Mann–Whitney U tests. Correlations were assessed by two-tailed Spearman test. Scatter plots and Bland–Altman plots with 95% limits of agreement were constructed.

## Results

### Patients and general information

**Fig. 1:**
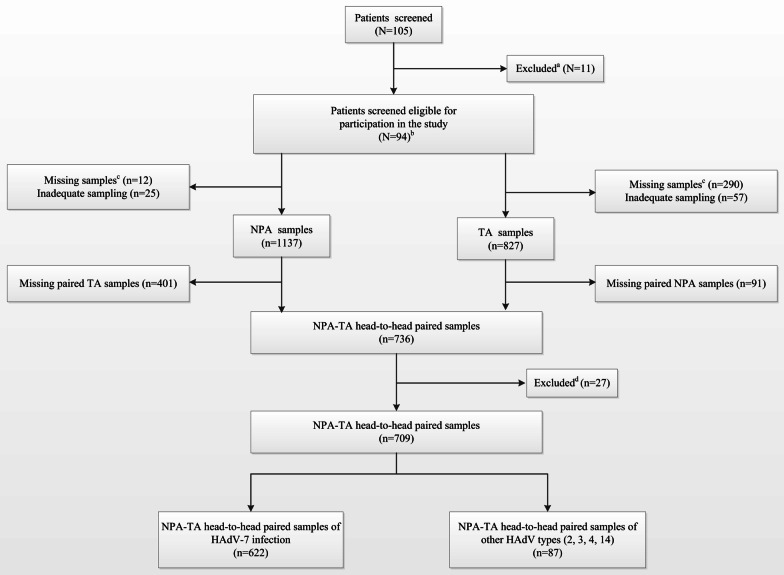
Flow-chart of patient enrolment. ^a^: One patient had undergone invasive ventilation for less than 3 days (patient deceased); four patients were unsuccessfully typed; and two children's guardians refused to provide paired TA specimens. Four patients had positive NPA samples, but four consecutive negative TA samples. ^b^: Among specimens from 94 patients, phylogenetic analysis classified 78 as HAdV-7, 12 as HAdV-3, 2 as HAdV-2, 1 as HAdV-4, and 1 as HAdV-14. ^c^: Reasons for samples included patients being unsuitable for sampling due to a critical condition, insufficient mucus in the nasopharynx or trachea, and sample not collected. ^d^: Twenty-seven pairs of NPA-TA samples were excluded, including eight that were both negative and nineteen that consisted of a positive and negative sample (positive sample viral load range: 2.64–8.67 log10 copies/mL; median = 5.07 log10 copies/mL). In 12 of the latter 19 sample pairs, the TA sample was positive, and the NPA sample was negative. The paired samples collected before and after each of the 27 excluded paired samples were positive for HAdV DNA, indicating that the negative results were due to experimental error. Abbreviations: N, number of patients; n, number of samples; NPA, nasopharyngeal aspirate; TA, tracheal aspirate

A total of 105 patients were enrolled. Eleven patients were excluded, including four with an HAdV DNA load < 1 × 10^2^ copies/mL in TA samples for 4 consecutive days after invasive ventilation (6.34 × 10^2^, 2.59 × 10^2^, 1.31 × 10^2^, and 2.05 × 10^2^ copies/mL, respectively, in initial NPA testing); one with < 3 days of invasive ventilation (deceased); two whose guardians refused continued sampling; and four with an unknown HAdV type. Thus, 94 patients were enrolled in the study (Fig. 1) and provided 736 simultaneously obtained paired NPA-TA samples. Twenty-seven pairs of NPA-TA samples were excluded. Thus, 709 pairs of positive samples were included in the study.

**Fig. 2:**
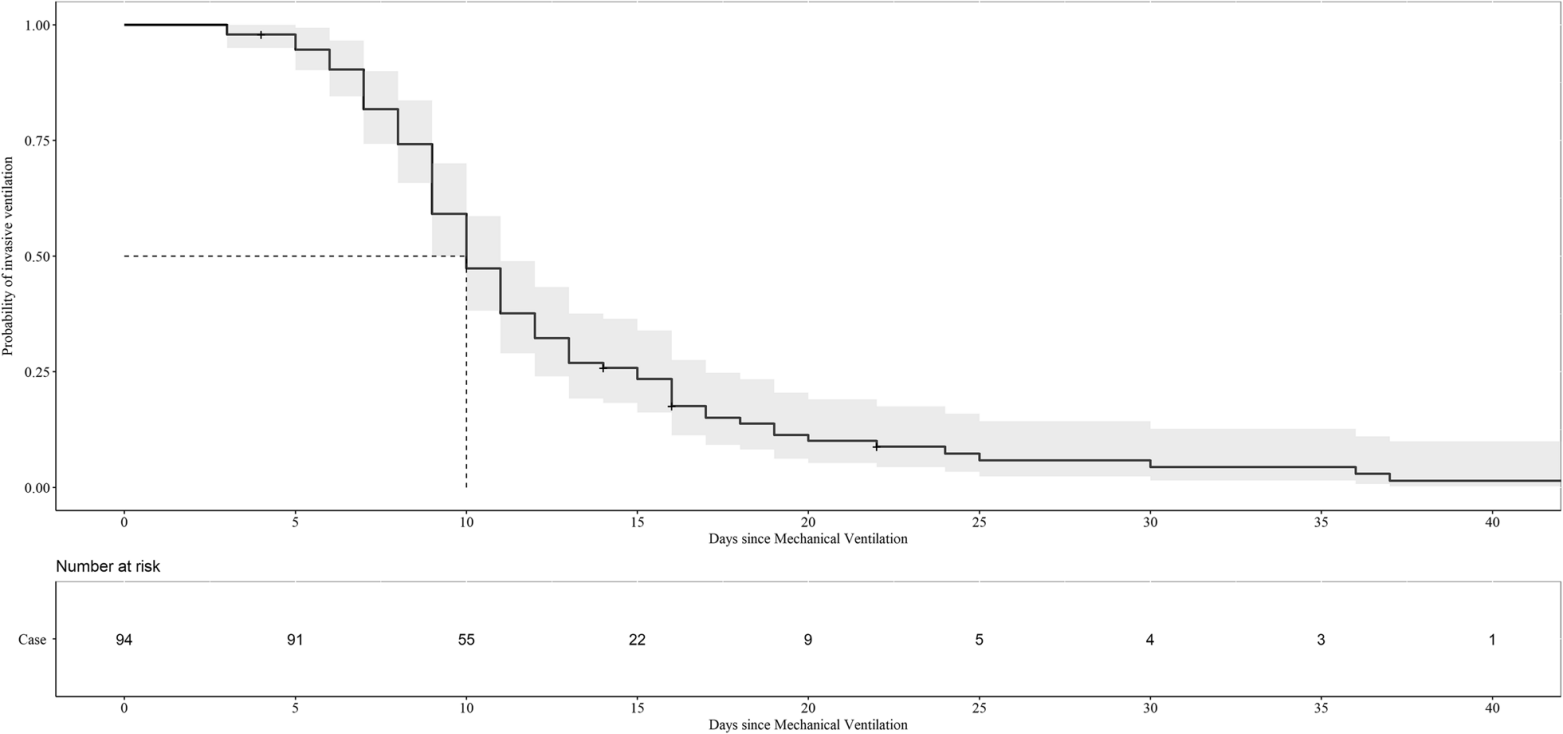
Kaplan–Meier curve showing the proportion of invasive ventilation

Of the 94 children studied, the ratio of boys to girls was 2.13:1. Male preponderance was most prominent with regard to infection with HAdV. The median age of the children was 12.0 months (IQR: 11.0–24.0), and > 90% of patients were aged between 6 and 59 months. The common genotypes in 94 cases were HAdV-7 (78/94, 82.98%) and HAdV-3 (12/94, 12.77%). Among 94 HAdV-infected patients, 72 were infected with HAdV alone and 22 (23.40%) had coinfections. Four patients were positive for both HAdV and parainfluenza 3, and one patient was positive for HAdV and influenza A. Three children were infected with HAdV, parainfluenza 3, and *Mycoplasma pneumoniae*. Fourteen children were infected with HAdV and *M. pneumoniae*. The median interval from onset to hospitalization was 6 days (IQR, 4.00–8.25). All patients had fever and cough (rectal temperature ≥ 38 °C or axillary temperature ≥ 37.5 °C). The median peak temperature was 39.8 °C, and the median duration of fever was 18 days. Together with fever and cough, wheezing (32.98%) was a common respiratory symptom. Gastrointestinal symptoms included diarrhoea (8 [8.51%]) and vomiting (10 [10.64%]). The median duration of mechanical ventilation and length of stay in the PICU were 10 and 17.0 days, respectively. We used a Kaplan–Meier plot to assess the cohort size over time as patients were extubated (Fig. 2). All patients received glucocorticoids and gamma-globulin therapy, and none received antiviral treatment. All five children who died were infected with HAdV-7, and one patient had underlying diseases (Table 1).

**Table 1:** Clinical characteristics of invasively ventilated hospitalized children with human adenovirus pneumonia

Characteristics	All patients	HAdV-7 infected	Other HAdV types infected	Single HAdV-7 infected	Single other HAdV types infected
	(n = 94)	(n = 78)	(n = 16)	(n = 59)	(n = 13)
	No. (%)	No. (%)	No. (%)	No. (%)	No. (%)
*Gender (male)*	64 (68.09)	50 (64.10)	14 (87.50)	36 (61.02)	12 (92.31)^b^
*Age (months), median (IQR)*	12 (11.00, 24.00)	12 (10.75, 24.00)	12 (11.25, 33.50)	12 (11.00,24.00)	12 (9.00, 25.00)
< 6	3 (3.19)	2 (2.56)	1 (6.25)	1 (1.69)	1 (7.69)
6–11	26 (27.66)	23 (29.49)	3 (18.75)	17 (28.81)	3 (23.08)
12–35	49 (52.13)	41 (52.56)	8 (50.00)	34 (57.63)	7 (53.85)
36–59	12 (12.77)	9 (11.54)	3 (18.75)	6 (10.17)	2 (15.38)
≥ 60	4 (4.26)	3 (3.85)	1 (6.25)	1 (1.69)	0
*Clinical features*					
Duration of onset (days), median (IQR)	6.00 (4.00, 8.25)	6.00 (4.00, 10.00)	5.50 (4.00, 8.00)	6.00 (4.00, 7.00)	5.00 (4.00, 7.00)
Fever	94 (100.00)	78 (100.00)	16 (100.00)	59 (100.00)	13 (100.00)
Tmax (°C), median (IQR)	39.8 (39.5, 40.1)	39.8 (39.5, 40.0)	39.8 (39.2, 40.3)	39.80 (39.50,40.00)	39.70 (39.40, 40.30)
Duration of fever (days), median (IQR)	17.50 (13.00, 24.00)	19.00 (13.75, 25.00)	15.50 (11.25, 17.00)^a^	19.00 (14.00,25.00)	15.00 (11.00, 18.50)^b^
Cough	94 (100.00)	78 (100.00)	16 (100.00)	59 (100.00)	13 (100.00)
Wheezing	31 (32.98)	28 (35.90)	3 (18.75)	24 (40.68)	4 (30.77)
Diarrhea	8 (8.51)	7 (8.97)	1 (6.25)	7 (11.86)	1 (7.69)
Vomiting	10 (10.64)	10 (12.82)	0	9 (15.25)	1 (7.69)
*Underlying diseases*	3 (3.19)	2 (2.56)	1 (6.25)	3 (5.08)	0
BPD	1 (1.06)	1 (1.28)	0	1 (1.69)	0
Hemodynamically significant heart disease	2 (2.13)	1 (1.28)	1 (6.25)	2 (3.39)	0
*Clinical outcomes*					
Duration of onset to intubation (days), median (IQR)	8.00 (7.00, 9.00)	8.00 (7.00, 10.00)	7.00 (6.00, 8.00)	8.00 (7.00, 10.00)	7.00 (6.00, 7.50)^b^
Duration of mechanical ventilation (days), median (IQR)	10.00 (8.00, 14.00)	10.50 (9.00, 14.25)	8.50 (7.00, 11.00)^a^	11.00 (9.00, 15.00)	9.00 (7.00, 13.00)
LOS in PICU (days), median (IQR)	17.00 (13.00, 23.00)	17.00 (14.00, 23.00)	13.00 (10.00, 18.00)^a^	17.00 (14.00, 23.00)	14.00 (12.00, 18.00)^b^
LOS in hospital (days), median (IQR)	26.00 (20.00, 33.00)	27.00 (21.00, 33.00)	19.50 (17.00, 25.00)^a^	27.00 (20.00, 33.00)	24.00 (17.50, 25.00)^b^
Death	5 (5.32)	5 (6.41)	0	4 (6.78)	0
Co-infected	22 (23.40)	19 (26.39)	3 (13.64)		

Other HAdV types of children, 12 were HAdV-3, 2 were HAdV-2, 1 was HAdV-4, and 1 was HAdV-14. n indicates the patient’s number. LOS indicates the length of stay. PICU indicates pediatric intensive care unit. BPD indicates bronchopulmonary dysplasia

HAdV-7 infected group is the children infected by HAdV-7. Other types infected group is the children infected by adenovirus types 2, 3, 4, 14. Single HAdV-7 infections and single other types infections group are the children only infected by HAdV-7 or other HAdV types. Continuous variables were described by median and 25th and 75th interquartile range (IQR). Comparisons between types of infection, as defined by these groups, were made using χ2 tests or the Fisher exact test as appropriate for categorical variables and the Wilcoxon rank-sum test as appropriate for continuous variables

^a^*P* < 0 .05, for comparison of HAdV-7 infected to other HAdV types infected

^b^*P* < 0.05, for comparison of single HAdV-7 infected to single other HAdV types infected

There were 19 cases of coinfection in the children in the HAdV-7-infected group and 3 cases in the other HAdV-type-infected group. The age distribution and clinical symptoms were generally similar among the groups (Table 1). Compared with children infected with other HAdV types, those with HAdV-7 had longer duration of fever and mechanical ventilation and longer stays in the PICU and hospital. All of the deaths occurred in type-7 infections. After excluding the influence of coinfection, patients infected by HAdV-7 alone had a longer interval from onset to intubation, longer duration of fever, and longer length of stay in the PICU and hospitals than patients infected by the other HAdV-type alone. However, there was no difference in the duration of mechanical ventilation between the two groups.

### Correlation of viral load between TA and NPA samples

We studied 709 pairs of respiratory samples collected from 94 individuals at different stages of infection. The viral loads ranged from 2.37 to 11.08 log10 copies/mL; the median values for the NPA and TA samples were 7.31 log10 and 7.50 log10 copies/mL, respectively. The median viral loads after 1, 2, 3 and > 3 weeks of the disease course was 8.65, 7.70, 6.69, and 5.09 log10 copies/mL, respectively, in NPA samples and 8.67, 7.79, 7.08, and 5.53 log10 copies/mL, respectively, in TA samples. Viral load was significantly higher in TA than NPA samples (Wilcoxon, Z =  − 2.281, *P* = 0.023).

**Fig. 3:**
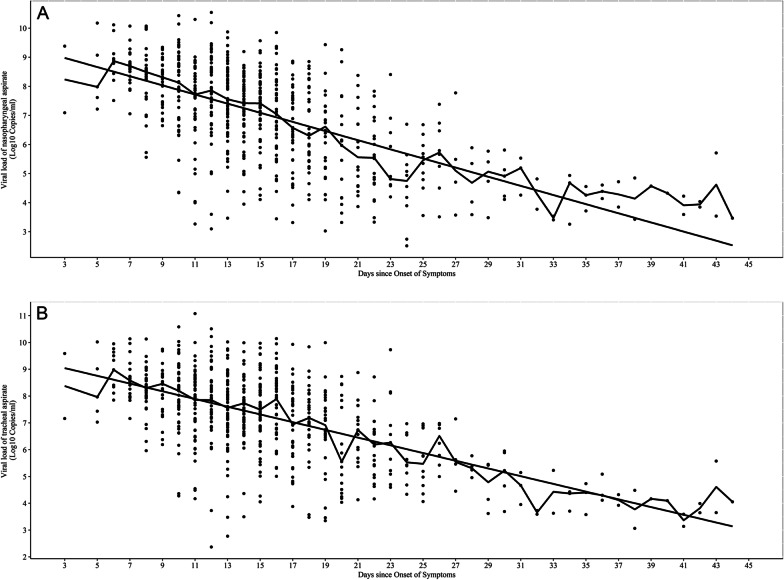
Linear regression showing the variation in viral loads of severe pneumonia after fever onset in NPA and TA samples from 94 children with HAdV pneumonia. A, Viral shedding in NPA samples; B, Viral shedding in TA samples. Abbreviations: NPA, nasopharyngeal aspirate; TA, tracheal aspirate

We monitored the kinetics of HAdV viral loads in upper and lower respiratory specimens from 94 patients. We analysed 709 NPA (Fig. 3A) and 709 TA (Fig. 3B) samples; there were > 3 sequential paired samples for each patient. There was a significant negative correlation between viral load and time since onset in both NPA samples (Spearman r =  − 0.607, *P* = 0.000) and TA samples (Spearman r =  − 0.544, *P* = 0.000). The viral load reduction rate and predicted duration of shedding were − 0.157 log10 copies/mL per day (95% confidence interval [CI]: − 0.143 to − 0.171; R^2^ = 0.397) and 60.17 days (y = 9.447 − 0.157x), respectively, for NPA and − 0.144 log10 copies/mL per day (95% CI: − 0.13 to − 0.158; R^2^ = 0.368) and 65.81 days (y = 9.476 − 0.144x), respectively, for TA. Pre-extubation viral loads (*i.e.*, the last paired samples for each patient) were also similar in the same patients. The median viral loads of NPA and TA were 5.55 log10 copies/mL (IQR: 5.01–5.95) and 5.74 log10 copies/mL (IQR: 5.06–6.37), respectively. Furthermore, all patients remained virus-positive in the invasive ventilation stages of infection. Severe cases had prolonged viral shedding in respiratory secretions, *i.e.*, viral DNA was still detected in five patients > 30 days after the onset of symptoms.

**Fig. 4:**
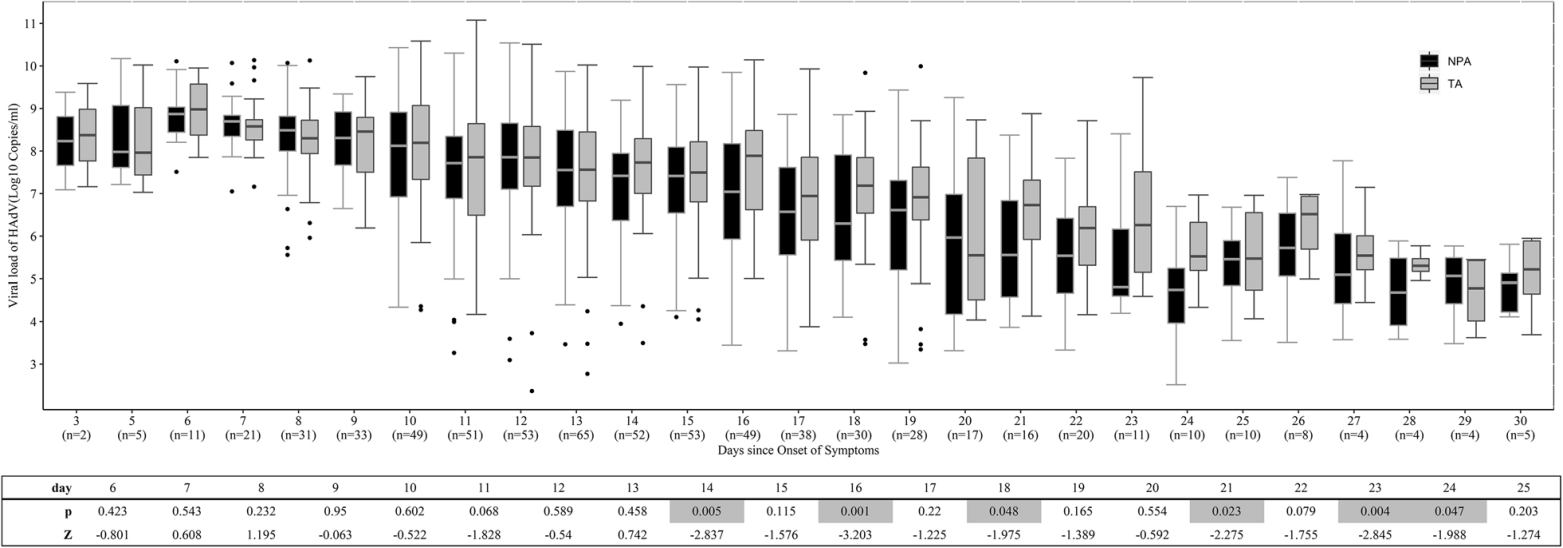
Viral loads (median [IQR]) in NPA and TA samples collected from 94 patients at days 3–30 after disease onset. The data after day 30 of onset were not due to small numbers of paired samples. Continuous data for > 10 paired samples were analysed by 2-tailed paired Mann–Whitney U tests. All *P* values indicate significant differences. Abbreviations: n, number of pairs of samples; NPA, nasopharyngeal aspirate; TA, tracheal aspirate

**Fig. 5:**
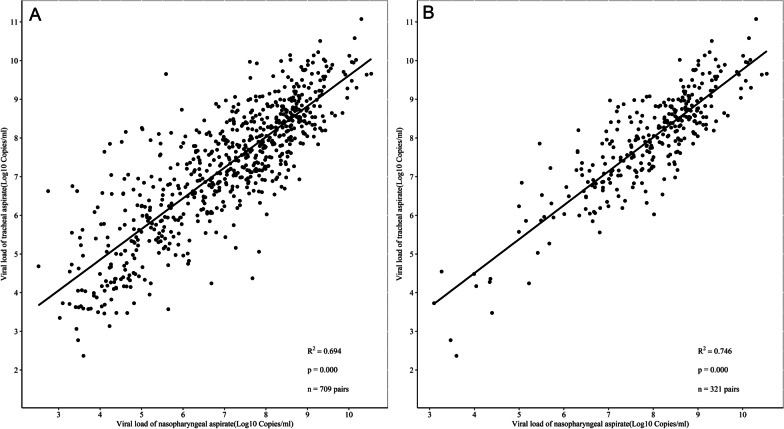
Correlation of viral loads between NPA samples and TA samples. Solid line represents linear correlation. **A** Viral loads of 709 pairs of NPA-TA samples over the course of the disease (R^2^ = 0.694, *P* = 0.000). **B** Viral loads of 321 pairs of NPA-TA samples 6–13 days after disease onset (R^2^ = 0.746, *P* = 0.000). Abbreviations: n, number of pairs of samples; NPA, nasopharyngeal aspirate; TA, tracheal aspirate

**Fig. 6:**
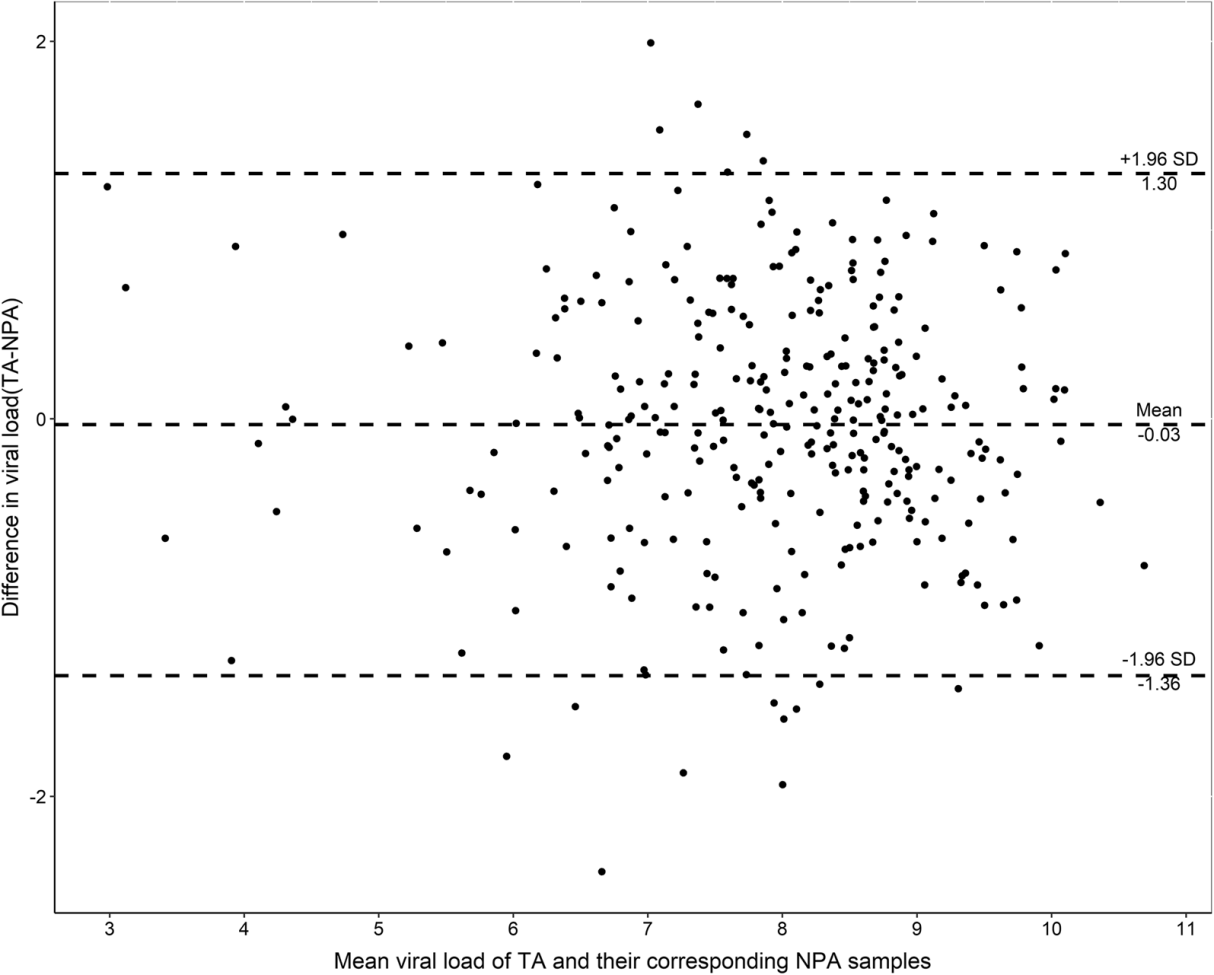
Bland–Altman plot displaying the difference in HAdV DNA loads between 321 paired positive NPA and TA samples obtained 6–13 days after disease onset. Dotted lines represent 95% limits of agreement (− 1.36 and 1.30 log10 copies/mL). Abbreviations: NPA, nasopharyngeal aspirate; TA, tracheal aspirate

We further plotted viral loads (median [IQR]) for NPA and TA samples collected from 94 patients at different disease stages. There was no significant difference in viral loads between NPA and TA samples at days 6–13 after onset, but variation increased during the late phase of infection (days 14, 16, 18, 21, 23, and 24), with loads remaining significantly higher in TA than NPA samples (Fig. 4). The viral loads were significantly correlated between NPA and TA samples (R^2^ = 0.694, *P* = 0.000) (Fig. 5). Cooccurring viral loads from NPA and TA samples of the same subjects also correlated well with each other. Viral loads of TA and NPA samples from the 321 pairs available from days 6–13 correlated significantly (R^2^ = 0.746, *P* = 0.000) (Fig. 4). The 95% limits of agreement for paired samples from days 6–13 were − 1.36 and 1.30 log10 copies/mL (Fig. 6).

## Discussion

We found that fever and cough were common clinical presentations in 94 mechanically ventilated children (median age = 12 months) with HAdV pneumonia. HAdV-7 was the main infection genotype, and all of the deaths were in HAdV-7-infected patients. We observed that HAdV-7 infection has a worse outcome than other HAdV types of infection, as others have reported [5, 7, 24–26]. In the present series, outcome measures were similar for the mixed group compared to the pure human adenovirus group (data not shown). Interestingly, we found that children with single HAdV-7 infections had a longer duration of fever, PICU stays, and hospitalization than the other HAdV-type alone infections group. However, there was no significant difference in the interval of mechanical ventilation between the two groups, suggesting that mixed infection may influence the duration of mechanical ventilation. In general, polymicrobial infections in community-acquired pneumonia have been considered to cause more severe inflammation and clinical disease than single microbial infections [27–29]. In some previous studies, the presence of viruses or atypical bacterial pathogens also did not appear to affect outcomes [30, 31]. The small number of patients in the present study prevents robust conclusions, considering the importance of coinfections on the outcome.

We found that the variation in viral loads in NPA and TA samples was similar and presented relatively high at the early stages of the disease and gradually decreased thereafter. Similar results were observed in adult patients with HAdV-55, where the viral loads were highest in the earliest sputum or nasopharyngeal swab samples (8.69 log10 copies/mL) [32]. In the present study, the median viral loads were highest in the first week of disease (8.65 and 8.67 log10 copies/mL, respectively, in NPA and TA samples). This prompted antiviral therapy to be initiated as early as possible. In addition, HAdV shedding in respiratory specimens persisted for > 2 months, and the predicted shedding durations were similar between NPA and TA samples. Ganime et al*.* reported that HAdV has a long survival time and can contaminate the hospital environment [33]. Increased monitoring of viral shedding may help reduce the spread of infection, especially for children in intensive care units undergoing high-risk surgery. The pattern of viral shedding in the present study was similar to that reported previously in adults [32, 34]. Huh et al*.* reported that the viral loads of HAdV-55 declined consistently in a log-linear fashion at a rate of − 0.15 log10 copies/mL per day, and a regression model estimated the viral shedding duration to be 55 days [32]. The rate of viral load decline was also similar to our study, but the duration of shedding was slightly shorter. However, our estimations were limited by the fact that most patients were intubated for < 2 weeks.

Viral loads in paired NPA-TA samples were significantly correlated in this study. Normalization of viral loads to the level of a housekeeping gene (glyceraldehyde-3-phosphate dehydrogenase) did not reduce the observed variability (data not shown). We also found that viral loads correlated significantly during the initial phase of infection (6–13 days after onset), with NPA samples explaining nearly 75% of the variance in TA samples. A previous study that included all ages found similar consistency between NPA and bronchoalveolar lavage fluid using a chemical direct immunofluorescence assay [19]. An aetiological study of Australian children with lower respiratory tract infection used PCR to show that HAdV positivity in nasopharyngeal swabs was associated with lower respiratory tract infection [20]. NPA samples can be acquired less invasively and used to monitor viral shedding trends. The present study showed that for children with HAdV pneumonia, the viral loads in NPA and TA samples were significantly correlated, especially at days 6–13 of the disease course. We recommend that patients be sampled, including collecting NPA, as early as possible during the course of infection to obtain the most reliable measure of HAdV.

We found that the variation in viral loads increased during the late phase of infection, with loads being significantly higher in TA than NPA samples. Previous studies have shown that virus replication and inflammatory responses differ among various parts of the airways and lungs [35, 36]. Therefore, the difference in viral loads between TA and NPA samples during the late phase of infection may be related to the pathogenesis of adenoviral pneumonia.

We also found that the pre-extubation viral loads (*i.e*., the last paired samples for each patient) were similar among all patients. To improve prognosis and disease management, the association between viral loads and ventilator weaning should be assessed in a randomized controlled trial with a process evaluation (such as by an ethics committee).

Our study had several limitations. First, it was a single-centre cohort study, and we did not enrol patients with early-stage or mild HAdV pneumonia or those who did not require invasive ventilation or were asymptomatically healthy; this was due to ethical considerations. Thus, there is some uncertainty as to whether our results can be extended to mild HAdV pneumonia. Additionally, viral loads are influenced by many factors. Although the samples in this study were collected by experienced nurses and sent for testing by specialized laboratory staff, confounding human factors may still have affected the sampling process. Viral shedding was also affected by factors such as genotype and therapy (data not shown). Furthermore, viral cultures from the collected samples were not seeded. PCR has high sensitivity, is easy to perform, and is widely used to detect viral loads. In our patients, continuous monitoring of HAdV load was not consistent with transient shedding during latent infection or transient shedding of residual virus. Finally, although multiple respiratory tract samples were collected from each patient for virus testing, we did not determine the precise timing of the “turning point” (*i.e*., from positive to negative virus detection).

## Conclusions

In conclusion, viral loads in both NPA and TA steadily decreased with the course of the disease, and the duration of HAdV shedding in respiratory specimens was long. The HAdV DNA load of NPA is highly correlated with that of TA, especially in the initial phase of infection.

## Data Availability

The datasets of the current study are available from the corresponding author on reasonable request.

## Abbreviations

HAdV: Human adenovirus
NPA: Nasopharyngeal aspirates
TA: Tracheal aspirates
PICU: Pediatric intensive care unit
IQR: Interquartile ranges
CI: Confidence interval
